# Sex Pheromone Aerosol Devices for Mating Disruption: Challenges for a Brighter Future

**DOI:** 10.3390/insects10100308

**Published:** 2019-09-20

**Authors:** Giovanni Benelli, Andrea Lucchi, Donald Thomson, Claudio Ioriatti

**Affiliations:** 1 Department of Agriculture, Food and Environment, University of Pisa, via del Borghetto 80, 56124 Pisa, Italy; giovanni.benelli@unipi.it; 2 Pacific Biocontrol Corporation, 14615 NE 13th Court Suite A Vancouver, WA 98685, USA; dthomso123@mac.com; 3 Technological Transfer Centre and Research and Innovation Centre, Fondazione Edmund Mach (FEM), via E. Mach 1, 38010 San Michele all’Adige (TN), Italy; claudio.ioriatti@fmach.it

**Keywords:** beetle pests, integrated pest management, mealybugs, moth pests, insect sex pheromones, precision agriculture

## Abstract

Pheromone-mediated mating disruption (MD) represents an important tool to manage insect pests in agriculture and forestry. MD relies on the release of synthetic sex pheromones from dispensers in crops, interfering with mate finding and reproduction of a pest through both competitive and non-competitive mechanisms. MD programs primarily rely upon “passive” dispensers, used at high densities per hectare (200–3000 units∙ha^−1^). In addition to the labor required for their application, another disadvantage of “passive” dispensers is the continuous release of pheromones, regardless of the time of day or the pest flight activity. Aerosol delivery systems can overcome the drawbacks of passive dispensers as they are applied at far lower density (2–5 units∙ha^−1^) and they can be programmed to release pheromones at selected time intervals when the target pest is active. However, the mode of action of aerosol dispensers is still not well understood and there are concerns of whether they are as effective as passive dispensers. This review focuses on the history of aerosol dispensers, mode of action, and effectiveness on various crops; deployment strategies; and the movement of pheromone once released. Limitations of aerosols and challenges for future research and commercial use are discussed.

## 1. Introduction

Currently, the European Commission Directives and Regulations are strongly encouraging a major reduction in pesticide use [1], with the aim of boosting the production of residue-free foods and reducing the negative impacts of pesticides on human health and the environment [2,3]. With this goal, the development of effective and environmentally sustainable integrated pest management (IPM) tools and strategies to manage insect pest populations is a key challenge [4]. Pheromone-mediated mating disruption (MD) is and will continue to be an important tool to help achieve the goal of the European Commission. Indeed, MD does not negatively impact non-target organisms, making this method fully compatible with the goals of Integrated Pest Management (IPM) [5,6,7,8].

Worldwide, agricultural pests on more than 800,000 hectares are estimated to be managed with MD. Most of these insect pests are in the order of Lepidoptera and the only two examples of pest belonging to other orders that are controlled with commercial formulation of MD are the vine mealybug *Planococcus ficus* Signoret (Hemiptera, Pseudococcidae) and the California red scale *Aonidiella aurantii* (Maskell) (Hemiptera: Diaspididae). In the European Union (EU), about 300,000 ha of vineyards are treated with MD to control the European grapevine moth, *Lobesia botrana* (Denis & Schiffermüller) (Lepidoptera: Tortricidae), including 90,000, 84,000, 60,000, and 43,000 ha in France, Spain, Germany, and Italy, respectively. A recent case study of high-valued vineyards in coastal Tuscany, Italy, demonstrated the cost-effectiveness of area-wide MD for *L. botrana* on more than 1000 ha [4]. In a recent US survey, Brunner [9] reported that 90% of pome fruit growers in Washington State, USA use MD in their insect pest management programs. A comparable percentage of apple orchards in Trentino-South Tyrol (Northern Italy) is currently managed through MD [8].

MD relies on the release of synthetic sex pheromones to interfere with mate finding and reproduction of target insect pests [10,11,12,13]. For field application, pheromones need to be formulated to prevent environmental degradation and to control their release into crop canopies [14]. MD can be achieved by non-competitive and competitive mechanisms [15]. Non-competitive mechanisms include camouflage, desensitization, and sensory imbalance. Competitive mechanisms relate primarily, but not necessarily exclusively, to false plume following [13,15].

## 2. Why We Need Aerosol Devices for MD Programs

Over the last 30 years, many dispenser technologies have been developed for pheromone-mediated MD [8,15,16,17]. Most technologies developed and commercialized have been “passive” dispensers that continuously release sex pheromones in response to physical parameters, regardless of the time of day or the flight activity of the target insects; thus their pheromone emission rates are guided mainly by temperature, pheromone volatility, and the design of the dispenser. For example, adult codling moth *Cydia pomonella* (L.) (Lepidoptera: Tortricidae), a major pest of pome fruit, are biologically active only near dusk yet about 90% of the pheromone applied is released outside the diel flight period [6,18]. Other limitations of “passive” dispensers include the availability and cost of labor for deployment and the inability to properly deploy dispensers in crops with large canopies, such as walnuts [19], or in field crops without canopies, such as rice [20].

MD with aerosol formulations is achieved with the application of widely spaced pheromone dispensers. The “transition” step between “passive” and aerosol dispensers are the so-called “large evaporators” or meso dispensers [5]. Early studies by Farkas et al. [21] evaluated the deployment of large evaporators containing *cis*-7-dodecenyl acetate (the single pheromone component of the cabbage looper moth, *Trichoplusia ni* (Hubner) (Noctuidae)) [22], in grids of 36, with the distance between devices of 200 or 400 m. With a pheromone release rate of 58 mg∙ha^−1^∙day^−1^, the authors observed a >95% disruption of communication (reduction of male orientation to female moths). Communication disruption with aerosols has also been tested in grape for control of the leafroller moth, *Platynota stultana* (Walsingham) (Tortricidae). Shorey et al. [23] reported that the deployment of 84 large evaporators per hectare in vineyards led to an MD performance similar to smaller dispensers (density: 252 units∙ha^−1^), with both dispensing systems providing the same daily release of pheromone ha^−1^.

“Active” release dispensers (i.e., aerosol devices) can overcome some of the limitations of passive and meso dispensers. For example, aerosol dispensers can be programmed to release sex pheromones at selected times and intervals when the target species is active [8] and they are faster and cheaper to apply. However, concerns exist of whether aerosol dispensers are as effective as passive dispensers due their wide spacing and the lack of knowledge on their mode of action in the field. There are other issues associated with aerosol devices, such as the disposal of the emitters, batteries, and cans.

In the following review, we will discuss the history of aerosol dispensers, mode of action and effectiveness, deployment strategies, and the movement of pheromone from aerosol dispensers. In the last section, we will discuss the limitations of aerosols, as well as the challenges for future research and commercial use.

## 3. History of Aerosol Devices for MD

Thomas et al. [24] first experimented with aerosol dispensers to disrupt the southern pine beetle *Dendroctonus frontalis* Zimmermann (Curculionidae), a pest of pine trees in southeastern USA. In 1996, a detailed description of an aerosol device used to disrupt *P. stultana* and *Spodoptera exigua* (Hubner) (Noctuidae) was provided and firstly named “puffer” [25]. The device was composed of an emitter and a pressurized canister loaded with the two-component pheromone blends of the two moth pests. The puffer was activated by light intensity and a timer programmed to release pheromone onto a piece of cloth fixed in front of the nozzle of the emitter acting as an evaporator. The puffers were spaced 40 m apart on the perimeters of 16-ha plots. The puffers completely shut down pheromone trap captures of *S. exigua* in tomatoes and *P. stultana* in grapes and peaches. The first publications by Shorey and coworkers about puffers focused only on the inhibition of moth captures [25,26,27], while later research by Elkins and Shorey [28] and Shorey and Gerber [29] demonstrated the efficacy of puffers to reduce crop damage in pears and tomatoes, respectively. During the same period, other aerosol technologies were being tested, including the metered semiochemical timed release system (MSTRS) [30,31] and the Michigan State University Microsprayer [32].

The success of aerosol technologies relied on precise and season-long release of insect pheromones without the need for maintenance, refilling, and component replacement. Aerosol technologies released the same total amount of sex pheromone per unit area as passive dispensers, but with from far fewer point sources, thereby relying on wind movement to disperse the pheromone through the crop [32].

In 1998, the Paramount Puffer^®^ PTB (Paramount Agricultural Technologies, Bakersfield, CA, USA) became the first commercial aerosol formulation to be registered by the US EPA (EPA registration number 71281-1) to control an insect pest, the peach twig borer *Anarsia lineatella* Zeller (Gelechiidae). Paramount soon followed up with registrations for codling moth, *C. pomonella*, and navel orangeworm, *Amyelois transitella* (Walker) (Pyralidae). Aerosol MD development and commercialization was restricted to the USA for almost a decade. The first area-wide (AW) use of aerosols was carried out in California from 1996 to 2002 to control codling moth. The project demonstrated the reliability and cost-effectiveness of aerosol dispensers [33]. The first commercial sales of aerosol MD outside of the USA was in Argentina in 2007 with the introduction of Puffer^®^ CM for the control of *C. pomonella* [34]. The Puffer^®^ system (at present commercialized by Suterra LLC (Suterra, Bend, Oregon, USA) was very similar to the puffers described by Shorey and Gerber [26] and Shorey et al. [25]. However, the Puffer^®^ consisted of a pressurized aerosol canister inside a programmed cabinet, without the addition of the evaporator cloth. Puffer^®^ technology has also been deployed at a density of 5 units∙ha^−1^ for MD of *A. transitella* in fig orchards in California [35]. Aerosol cans were loaded with (*Z,Z*)-11,13-hexadecadienal of ≥90% purity in either ethanol (2001 season) or a mixture of hexane + acetone (2002 season), emitting 0.2 mg of active ingredient (a.i.) every 15 min between 18:00 and 6:00. In the first European trials, Puffer^®^ CM (2 units∙ha^−1^) were successfully applied once during the 2005 season in two trials conducted in Northern Spain [36]. Aerosol dispensers have also been commercialized in South Africa, Italy, and Spain. In recent years, three other companies, Pacific Biocontrol Corporation (Vancouver, WA, USA), Semios Technologies Inc. (Vancouver, BC, Canada), and Scentry Biologicals, Inc (Billings, MT, USA), have commercialized aerosol MD products. The first products launched by these companies were for the control of *C. pomonella* in the USA, Isomate CM Mist in 2013, Semios CM in 2014, and Nomate^®^ CM Smart release in 2017, respectively [34] (Table 1).

## 4. Competitive and Non-Competitive Mechanisms

According to Miller and Gut [15], the mode of action of MD occurs by either competitive or non-competitive mechanisms. As described above, non-competitive attraction includes camouflage, desensitization, and sensory imbalance, while competitive attraction involves false-trail following [13,15,47]. However, there is a wide range of behavioral responses to MD specific to the physiological responses of the insect, its population density and distribution, and the dispensing technology [48,49]. In general, it has been observed that there is an increase in the MD efficacy with an increasing number of pheromone release points [8].

Passive dispensers release pheromone with a variable release rate relative to temperature. Pheromone dispensers are best distributed uniformly in the crop between 200 and 3000 dispensers per hectare. Aerosol emitters release small particles of pheromone actively, usually between 50 and 100 microns, and rely on wind movement to distribute the pheromone [50]. The release of pheromone by aerosol devices acts to delay mating and reduce the frequency of mate finding [19]. Among the modes of action discussed above, competitive attraction plays a major role in codling moth MD [49]. Males are attracted to dispensers and become exposed to high concentrations of pheromone, impacting the sensory structures either on the antennae or in the antennal lobe. No evidence has been found for camouflage being an important mechanism of codling moth MD [49]. However, if competitive attraction is the most important mode of action, then it stands to reason that passive dispensers deployed at a high density of point sources per hectare should be more efficacious than aerosol dispensers applied at two to five point sources per hectare.

The mechanisms of MD with aerosol dispensers has been investigated by a number of authors. Aerosol devices release large plumes of pheromone great distances downwind from the source point to disrupt moth communication over large areas [51]. However, it is also conceivable that sex pheromone emissions from widely spaced aerosol devices could mimic the plumes emitted by females of the target insect at great distances downwind. On the other hand, at least in close proximity of the aerosol emitter, high-dose exposures to pheromone from aerosol devices may result in habituation, leading to arrested movement [5]. Stelinski et al. [52] pointed out that the higher concentration of pheromone from aerosol devices may result in habituation to a greater degree than the low concentration of pheromone released from passive dispensers.

McGhee et al. [50], used both dose–response curves and quantitative tools developed by Miller et al. [48,49] to demonstrate that aerosol devices used for *C. pomonella* exert their MD action through a competitive mechanism. Indeed, *C. pomonella* moth captures decreased asymptotically as Isomate^®^ CM MIST densities increased, thus matching the predictions for competitive (i.e., false-plume following) rather than non-competitive MD (i.e., camouflage of females and traps). Isomate^®^ CM MIST has been found to be about 22 times more effective than a passive reservoir dispenser with respect to pheromone trap catch suppression [50]. However, a potential weakness of aerosol MD is that the low density of emitters may result in areas of the crop with poor pheromone coverage [50]. Therefore, the application of aerosol-based MD on large and contiguous areas is a major requirement to ensure efficacy. McGhee et al. [50] found that five MIST aerosols ha^−1^ provides the same level of control of codling moth as passive reservoir dispensers applied at 500 to 1000 dispensers∙ha^−1^. Both passive and aerosol dispensers act via the competitive attraction mechanism and as a result, the efficacy of both dispenser types is density dependent. Therefore, both MD technologies cannot achieve complete control when targeting high-density populations [50]. Codling moth research showed a little loss of efficiency with a decreased emission frequency [53], while the effectiveness of navel orangeworm mating disruption increases with emission frequency [54]. Possible factors leading to this difference include the chemical nature of the *A. transitella* pheromone compound involved (aldehyde moiety of the Z11,Z13-16:Ald has a potential to polymerize) and differences in the mechanism of disruption of sexual communication [54].

Pheromone released from aerosol devices has been shown to travel long distances. Shorey et al. [25] estimated that aerosol devices releasing 0.9 mg of pheromone components∙ha^−1^∙day^−1^ could be separated from each other and from centrally located female-baited traps by up to 100 m before communication disruption was substantially reduced. Welter et al. [5] conducted release-recapture experiments that demonstrated pheromone plumes greater than 460 m long and 90 to 150 m wide.

Pheromone sprayed from aerosols adheres on the leaves, thus pheromone molecules accumulate over time on the exposed surfaces of the tree [52]. Suckling et al. [55] stressed that moth sex pheromones can be absorbed and released by apple foliage in amounts that enhance disruption of catches of *Epiphyas postvittana* (Walker) (Tortricidae). The accumulation of pheromone may increase the efficacy of aerosol devices [55]. Based on field EAG results at 5 m downwind from an aerosol device, Suckling et al. [55] suggested that different plume structures may be linked to higher turbulence in the orchard canopy. These authors also observed that the percent reduction in moth captures obtained with aerosol devices in the New Zealand study was lower than in similar experiments conducted in the United States. The larger tree size and higher leaf area have been identified as potential factors reducing wind speed and therefore, the loss of pheromone in California orchards, thereby increasing efficacy compared to New Zealand orchards [55]. Research concerning the capture of male navel orangeworm moths, *A. transitella*, in female-baited traps positioned at five levels, from ground level to the canopy top, in almond orchards showed that the optimal height for the pheromone release by aerosol devices at low density (3 units∙ha^−1^) would be at mid or low height in the canopy, thus boosting pheromone dispersion throughout the canopy [56].

## 5. Effectiveness of Aerosol Devices for MD on Various Crops

The next sections will provide an overview of the use of aerosols in different cropping systems. Most research items focused on the reduction of moth catches, while only a limited number of studies evaluated the efficacy of MD aerosol devices in terms of crop damage reduction.

### 5.1. Fruit and Nut Trees (Apples, Pears, Peaches, Oranges, Plums, Figs, Almonds, Pistachios, Walnuts)

The first references to the use of aerosol-based MD to control moth pests of fruit trees were reported by Shorey and coworkers [25,26,27]. Successful area-wide MD programs using Puffer^®^ devices to manage *C. pomonella* in pome fruits were in California during the period 1995–2002 [33]. In California, until then, most of the MD devices deployed were passive dispensers (Isomate C+ rope dispensers and Checkmate CM laminate ones). During this early research, Puffer^®^ devices were successfully used on more than 520 ha of pears in Lake County [5]. The amount of pheromone in aerosol formulations was based upon the amount of pheromone contained in high-density passive dispensers deployed at 1000 units ha^−1^ [53].

**Figure 1:**
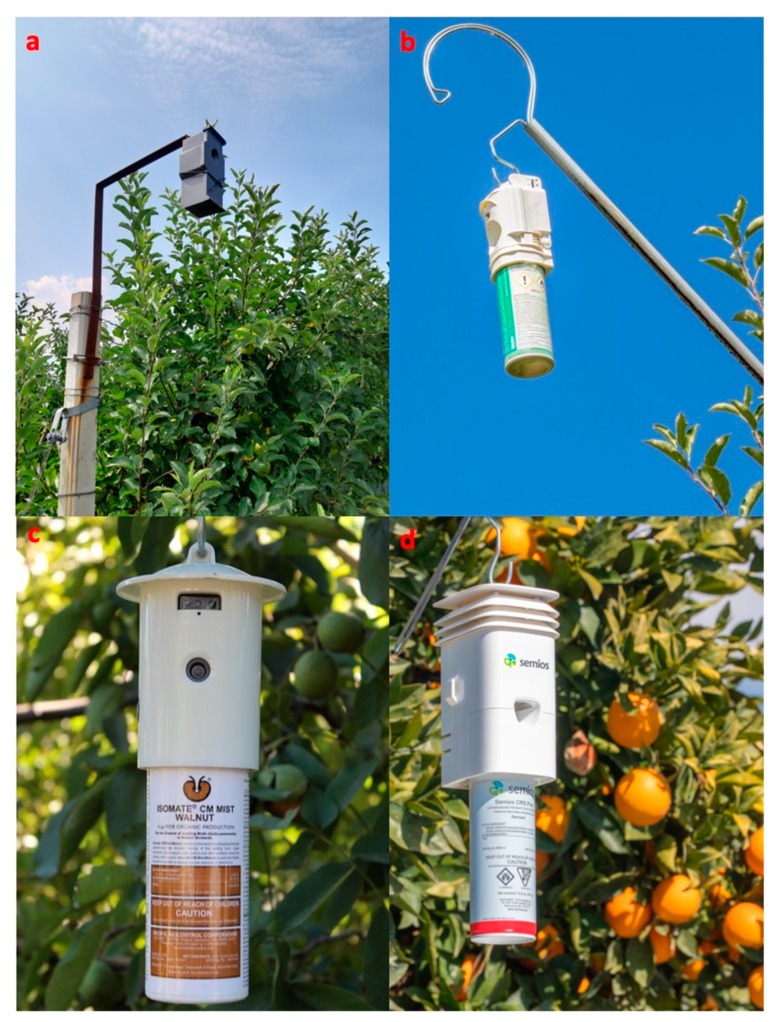
Examples of aerosol devices currently marketed (2018) for pheromone-mediated mating disruption of the codling moth, *Cydia pomonella*, in apple orchards, (**a**) CHECKMATE Puffer^®^ CM (Suterra), (**b**) ISOMATE^®^ CM MISTER (CBC Europe Srl-Italy) (photo credit: Claudio Ioriatti), (**c**) and in walnut orchards, ISOMATE^®^ CM MIST WALNUT (photo credit: Tina Phelps). (**d**) Management of *Aonidiella aurantii* in orange groves relying to Semios CRS Plus (Semios Technologies Inc. Canada) (photo credit: Kelly Petersen).

The size of the area that a single Puffer^®^ affects was estimated to be as much as a few hectares [5]. Notably, a valley shape in male catches (i.e., highly reduced or no captures downwind from the aerosol along the wind direction, coupled with increased captures upwind and/or to the sides of the study sites) was detected when a Puffer^®^ released pheromone in the environment. Puffer^®^ delays mating and reduces the frequency of mate finding by males [19]. Examples of aerosol devices currently marketed for their use in controlling the fruit pests *C. pomonella* and *A. aurantii* are shown in Figure 1.

From 2010 to 2013, the efficacy of Puffer^®^ CM was evaluated in Trentino (Northern Italy). It was shown that a single worker can apply Puffer^®^ CM on 3 to 5 hectares per hour. Results obtained with two aerosol units∙ha^−1^ were extremely promising, with a high level of catch suppression in the traps, a significant reduction of overwintering larval populations, and fruit damage levels comparable or even lower than the grower’s standard or passive releasing MD programs. In regions such as Trentino, where there are a large number of small fruit-producing farms, but strongly organized in producer associations, the application of aerosol devices can be more easily managed [57,58,59]. Research has shown that pheromone released from aerosol devices moves great distances downwind (i.e., from 100 to 200 m length and 70 m width) [5,57,58,59,60]. Casado et al. [19] showed that a single Puffer^®^ CM strongly reduced moth captures on several hectares, even at distances of over 300 m downwind.

**Figure 2:**
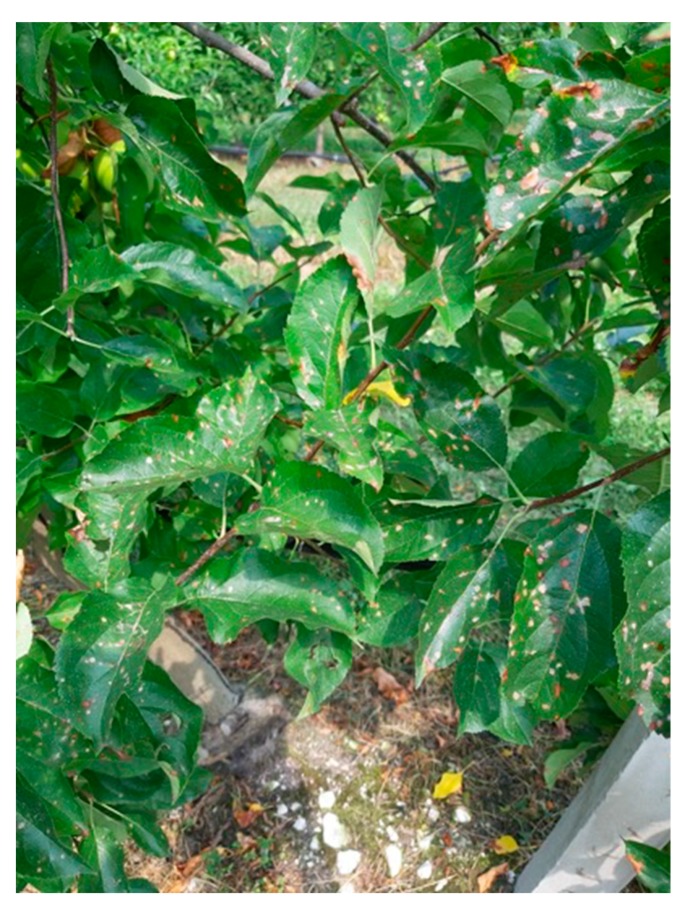
Codlemone phytotoxicity on leaves of the apple trees located in front of the aerosol device.

A disadvantage of CM aerosol devices can be phytoxicity. Pheromones are fatty-acid derivatives and can bind to compounds contained in plant tissues when they come into contact for a sufficient amount of time. The phenomenon is visually evident depending on the variety and in the presence of low temperatures that slow the evaporation of the pheromone deposited on the vegetable layer (Figure 2).

Giroux and Miller [61] reported that pheromones with a chain length of 6 to 13 carbons bearing a polar moiety (e.g., alcohol) are the most phytotoxic. Therefore, it is important how the codlemone [(8*E*,10*E*)-8,10-dodecadien-1-ol] pheromone emitters are placed in the tree. The authors stressed that pheromone-induced phytotoxicity was a minor problem, with growers failing to detect it in MD orchards on more of two trees per 0.4 ha [61].

In the early stage of aerosol technology development, the combination of aerosol devices with either passive dispensers or supplemental insecticide applications was also tested. Knight [62] developed a MD program (I HELP, Integrated High Emission Low Point) aimed at the simultaneous control of codling moth and leafrollers in orchards of 16 ha, located in Washington State. Aerosol devices have also been tested alone or coupled with passive dispensers on borders [62]. Knight [51] investigated the efficacy of deploying an internal grid of either aerosol dispensers or passive releasing dispensers clustered together and supplemented with border applications of passive dispensers for control of *C. pomonella*. Aerosol dispensers were applied at 2.5 Puffer^®^ units∙ha^−1^ and released 7 mg codlemone per day every 15 min from 17:00 to 05:00 h [51]. The author also reported effective codling moth MD using Puffers^®^ at 1 unit∙ha^−1^ in combination with a 10 to 20 m wide band of Isomate C Plus dispensers deployed at 1000 dispensers∙ha^−1^ in the border rows. Over 10 years, the use of aerosol devices in conjunction with judicious supplemental insecticides led to a decrease in broad-spectrum insecticide application and a subsequent decrease in pear psylla and mite populations [63].

In the process of adjustment of the use of aerosols for codling moth, Isomate^®^ CM MIST applied at 2.5 and 5 units∙ha^−1^ resulted in a trap catch inhibition of 83% and 91%, respectively [50]. The authors showed that the deployment of 5 Isomate CM MIST units∙ha^−1^ is needed to reach CM control levels given by “passive” reference MD products applied at 1,000 dispensers per hectare [50].

With respect to other fruit pests, it has been shown that Puffer^®^ aerosols at 5 units∙ha^−1^ significantly disrupted mating communication of *A. transitella* in 16-ha experimental plots within two separate fig orchards [35]. Also, trap catches of *E. postvittana* were 90% reduced in study sites treated with 5 aerosol devices∙ha^−1^ (Ecomist 7), while EAG assays outlined that a single pheromone plume is perceived as much as 40 m downwind in an orchard [55].

In Michigan and much of the eastern and midwestern USA, oriental fruit moth *Grapholita molesta* (Busck) (Tortricidae) is a key pest of apples, requiring simultaneous control with the codling moth [64,65]. Aerosol devices can be considered as a reliable and economically sustainable technology for MD of these two important moth pests, utilizing devices co-releasing the pheromone of both insects and deploying them at low densities (i.e., 2–2.5 units∙ha^−1^). Unfortunately, as reported by Stelinski et al. [52], aerosols used as a standing-alone control tool failed to achieve full control of codling moth (only 26% to 75% of MD based on trap capture suppression), while better results have been achieved for *G. molesta*, with efficacy rates reaching 84% to 98% of MD.

The Checkmate^®^ Puffer^®^ CM-O label, even with an application rate of 5 units∙ha^−1^, did not lead to optimal results in orchards <16 ha [53]. Puffers placed peripherally around 16-ha blocks and evenly spaced reduced male trap catches by >95% and female mating rates by >69%, with the best case performance achieved by evenly placed Puffer^®^. MD with gridded aerosols in almonds led to a limited reduction (i.e., <40%) of navel orangeworm, *A. transitella*, damage [66]. Earlier, on almonds, Mafra-Neto and Baker [30] showed that metered semiochemical timed release (MSTRS), a device with a design comparable to that of puffers spraying the pheromone, proposed by Shorey et al. [25], led to 100% MD of *Cadra cautella* Walker (Pyralidae) for 24 h, 92% after 72 h in trials conducted in 3 × 3 × 3 m cages with a 2:1 population of males and females.

Higbee et al. [67] investigated the effectiveness of aerosol devices for managing *A. transitella* in experimental plots within either large pistachios or almond orchards The aerosol devices at a density of 0.8 ha^−1^- released a combination of (11*Z*,13*Z*)-hexadecadienal, (11*Z*,13*Z*)-hexadecadien-1-ol, and (3*Z*,6*Z*,9*Z*,12*Z*,15*Z*)-tricosapentaene. The (11*Z*,13*Z*)-hexadecadienal is the major component of navel orangeworm sex pheromone, but by itself it is not attractive. However, in conjunction with the minor compounds (11*Z*,13*Z*)-hexadecadien-1-ol and (3*Z*,6*Z*,9*Z*,12*Z*,15*Z*)-tricosapentaene, the blend is very attractive. Field experiments carried out in 8-ha plots of pistachios showed >97% suppression of moth male catches, along with mating suppression rates in sentinel females ranging from 82% to 93%. Extremely promising results have also been achieved with the navel orangeworm formulation when applied in 16-ha plots of almonds, where male catches and mating were suppressed >99%. Notably, nut damage on Nonpareil (most common variety) almonds has been shown to be significantly reduced compared to almonds not treated with pheromone.

### 5.2. Grapes and Soft Fruits

**Figure 3:**
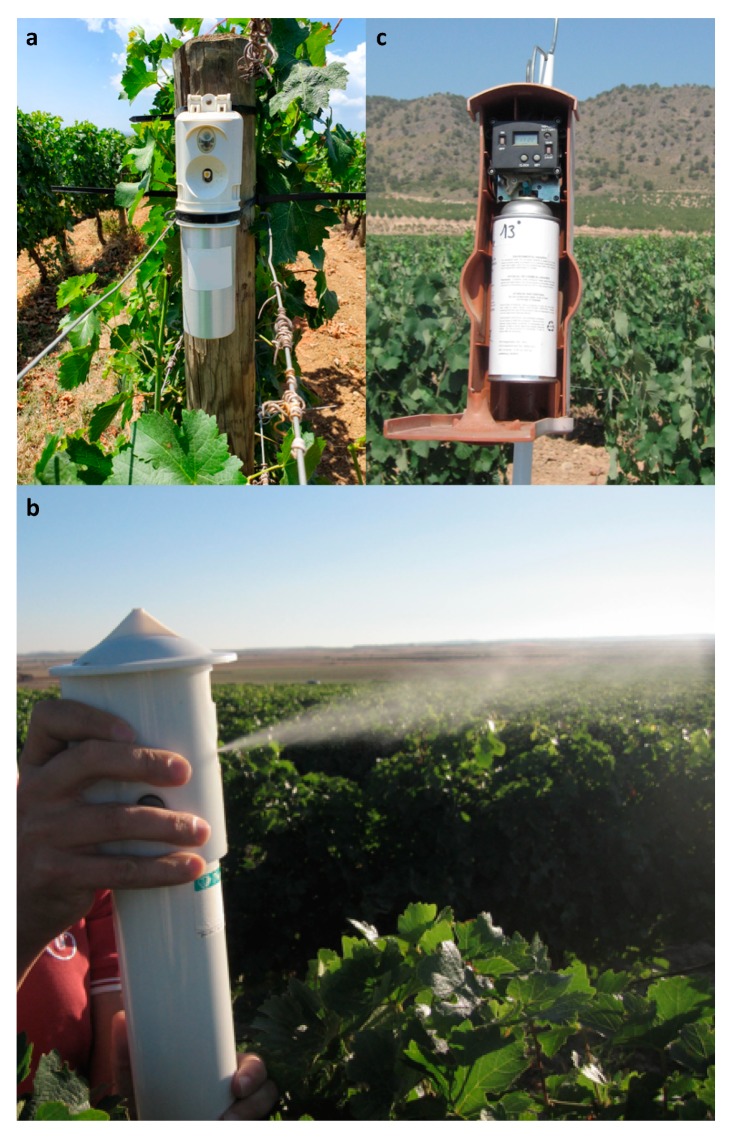
Aerosol devices used for pheromone-mediated mating disruption of the European grapevine moth, *Lobesia botrana*, on grapevine: (**a**) Isonet^®^ MISTER PRO L, (**b**) Isonet^®^ MISTER releasing a puff of *L. botrana* pheromone in a Spanish vineyard, (**c**) CheckMate^®^ Puffer^®^ LB (Suterra) (photo credit: Andrea Lucchi).

There is little published research on the use of aerosol devices to control insect pests of grape and soft fruits, such as cranberry. Shorey et al. [25] demonstrated the efficacy of aerosols to disrupt communication of *P. stultana* in grape. However, the impact on damage reduction has not yet been assessed. The first commercial aerosol formulation for European grapevine moth, *L. botrana*, was developed by Suterra in Spain. According to de Alfonso and Colás Roy [68], the Puffer^®^ technology (Figure 3) showed an equivalent efficacy to passive dispensers.

Lucchi et al. [42] evaluated the efficacy of an aerosol device, Isonet^®^ MISTER PRO L (Figure 3) releasing the main *L. botrana* pheromone component (i.e., (7*E*,9*Z*)-7,9-dodecadien-1-yl acetate) at selected time intervals. These authors compared the efficacy of Isonet^®^ MISTER PRO L at the density at two units∙ha^−1^ over two years in the Aragon region of Spain, with respect to the suppression of *L. botrana* catches, as well as larval infestation on bunches, to the MD product Isonet^®^ L and a grower’s standard. The deployment of Isonet^®^ MISTER PRO L significantly decreased male catches in MD plots over control ones. The number of infested bunches and nests per bunch was lower in MD plots compared to the grower’s standard. Isonet^®^ MISTER PRO L achieved comparable efficacy to the standard MD product Isonet^®^ L [42]. Recently, video camera-assisted pheromone traps were deployed to continuously monitor the flight of *L. botrana* over the course of a day [69]. Since most of the *L. botrana* flight occurred between dusk and midnight, the potential of finely tuning the time intervals of pheromone release, by concentrating aerosol releases during this period, is relevant and worthy of further research [69].

With respect to other soft fruits, Baker et al. [31] tested MSTRS™ devices for the MD of blackheaded fireworm, *Rhopobota naevana* (Hübner) (Tortricidae) in Wisconsin cranberry. During first flight, the aerosol devices were deployed either at 12 units∙ha^−1^ with three devices transecting the center of the study plot and the rest on the plot perimeter (i.e., cross pattern), and 5 or 12 devices ha^−1^ deployed around the perimeter of the study plot (i.e., perimeter patterns). Both treatments reduced male captures (10 μg lure) by >95% at two of the study sites. At a third study site, moth captures were reduced only by 81.7%, 80.7%, and 56.4% for the threetested patterns detailed above, respectively. During the second flight, pheromone was released only during the night, resulting in a reduction of mean trap catches >85% at two study sites, regardless from the tested deployment pattern, while in the third study site male catch reduction was only slightly higher than 50%. No significant catch differences were reported over the season with the different deployment patterns. The impact on larval populations was not significant; however, the authors claimed that this may be connected to high sampling variability and low infestation rates in the study plots, making any conclusion hard to draw [31]. Significant reductions in the mating frequency of free-flying *R. naevana* females by releasing 50 μg of pheromone per spray at 15-min intervals confirmed the potential of MSTRS™ devices for population control of this cranberry pest [43].

### 5.3. Field Crops (Artichokes and Tomatoes)

Shorey and Gerber [29] conducted pioneer research on the deployment of aerosol devices to control beet armyworm (*S. exigua*) season-long in tomatoes. Aerosol devices released pheromone at 46 mg ha^−1^ day^−1^. In total, 117 devices were spaced at 40 m around the perimeter of a 60-ha block and also distributed in the central zone. Shorey and Gerber [29] reported complete shutdown of moth captures in traps and a reduction in the abundance of egg masses and damage to the crop.

Bari [70,71] investigated the efficacy of 2,5 or 5 Puffer^®^ aerosol devices ha^−1^ to control the artichoke plume moth, *Platyptilia carduidactyla* (Riley) (Pterophoridae). Five aerosol devices ha^−1^ in conjunction with an insecticide program significantly reduced the pest damage on buds by ~50% [70]. Comparable results have been achieved by Shin-Etsu “rope” dispensers [70]. Further research conducted with the Puffer^®^ aerosol devices over a four-year period showed a bud damage reduction of about 60%. In addition, damage reduction was reported on nearby fields, showing the beneficial impact beyond the borders of treated plots. At the end of the four-year period, damage was reduced by 88% [70].

### 5.4. Stored Products (Dried Beans and Corn)

To the best of our knowledge, there are only two research publications on the efficacy of aerosol devices to manage stored product pests. Fadamiro and Baker [72] investigated the efficacy of aerosol MSTRS™ in corn storage rooms highly infested by the Indian meal moth, *Plodia interpunctella* (Hübner) (Pyralidae), and Angoumois grain moth, *Sitotroga cerealella* (Olivier) (Gelechiidae). MSTRS™ devices emitting ∼0.6 μg∙min^−1^ of [(*Z,E*)-9,12-tetradecyldienyl acetate, *Z*9,*E*12:14:Ac, for *P. interpunctella*] and ∼0.2 μg∙min^−1^ (*Z*7,*E*11-16:Ac for *S. cerealella*) disrupted pheromone moth captures by 70% and 40%, respectively. Mating of both species was reduced by 45% and ∼30%, respectively. Burks et al. [46] tested aerosol devices releasing 1.9 mg∙day^−1^ per 100 m^3^ of *Z*9,*E*12-14:Ac to manage *P. interpunctella* in stored beans. The aerosol treatment strongly reduced male catches in pheromone-baited traps, as well as the percentage of mated females. No female progeny were detected after two weeks of MD, indicating that the aerosol technology was highly effective in suppressing population growth of this key phycitine pest attacking stored products [Burks et al. 2011].

Fadamiro and Baker [72] and Burks et al. [46] used two different experimental devices to control stored product moths with aerosol mating disruption. The first article referred to the use of pressurized and non-pressurized versions of the MSTRS™. In this device, a two-component pheromone blend was emitted every 15 min (similar to many current commercial systems) and re-emitted from a pad (unlike current commercial systems). For the Michigan State Microsprayer reported by the second paper, pheromone (a single major component only) was emitted directly into the air every 2.5 min; much faster than current commercial systems. These differences may be relevant to the successful control of infestation in the case of Burks et al. [46] but not for Fadamiro and Baker [72]. However, the role of the different aerosol mating disruption systems in the different outcomes of these trials is complicated by the fact that the maize in the Fadamiro and Baker [72] trial was a more favorable host than the dried beans in the Burks et al. [46] trial.

### 5.5. Cereals, Forage and Fiber Crops

Fadamiro et al. [44] compared the efficacy of MSTRS™ devices and Shin-Etsu rope dispensers deployed at a density of 12 and 3000 units∙ha^−1^ respectively, in the grassy areas that serve as aggregation and mating sites for *Ostrinia nubilalis* (Hübner) (Crambidae) and from which mated females fly to adjacent cornfields. The study aimed at preventing females mating in these grassy areas in order to reduce egg laying and damage in nearby cornfields. The rope dispensers were deployed either as single dispensers 2 m apart (3000 units∙ha^−1^) or grouped in a widely spaced (35 m) pattern. Dispensers were loaded with a blend of (*Z*)-11-tetradecenyl acetate and (*E*)-11-tetradecenyl acetate (97:3), the sex pheromone of the European corn borer. Data showed that the MSTRS™ released pheromone at a rate 26-fold higher (6.09 μg∙min^−1^) than the Shin-Etsu rope (0.23 μg∙min^−1^). Both dispensers performed excellently during the first and second moth flight, achieving a 97% reduction in male moth captures. In addition, both treatments reduced the level of mating of wild females. During the first flight, the bursa copulatrix of wild females was examined to assess for the presence of and the number of spermatophores. The mean value was 1.33, 1.58, and 1.88 in the MSTRS™, rope, and untreated plots, respectively. During the second flight, the mean value was 1.63, 1.56, and 2.17 in the MSTRS™, rope, and untreated plots, respectively. The authors also reported a significant reduction (∼17%) in the proportion of females mating at least once during both flights in MSTRS™ plots [44]. More recently, Vacas et al. [20] conducted a study in a rice-growing area of Valencia (Spain) to investigate the efficacy of MD to control the striped rice stem borer, *Chilo suppressalis* Walker (Crambidae). The aerosol cans were either loaded with the complete pheromone blend of *C. suppressalis* (Neburel^®^-Z) or the major component, *Z*11-16:Ald (Neburel^®^-M). The aerosol devices were applied at 3 units∙ha^−1^. The efficacy of the aerosol devices was compared to two types of passive releasing dispensers: Saturel^®^ mesoporous dispensers and the conventional MD treatment with Selibate^®^CS. The passive releasing dispensers were deployed either at 5 or 10 release sites per hectare. At each site, dispensers were clustered at either 6 or 12 dispensers per release point. The aerosol dispensers released 6.6 to 7.9 g ha^−1^ and the 30 Selibate^®^CS dispensers∙ha^−1^ released ~5.0 g∙ha^−1^ of pheromone (i.e., values indicate total pheromone emitted during the study periods, from June to mid-September). The three MD treatments had comparable efficacy and the damage in all three treatments was less than 1% [20].

Mori and Evenden [73] conducted large-plot (5 ha) experiments to test the potential of aerosol devices, applied at the density of 2 units∙ha^−1^, to manage *Coleophora deauratella* Leinig and Zeller (Coleophoridae), in red clover seed production fields in Alberta, Canada. The research investigated the impact of the aerosol treatments to reduce male catches in pheromone traps and to assess larval abundance and the reduction in crop damage. Pheromone emissions were concentrated at sunrise, corresponding to the maximum peak of the male response to females’ pheromone. While aerosol-based MD achieved a strong reduction of male catches (∼94%), no significant reduction in larval populations nor increase in clover seed production were detected in MD plots, leading the authors to hypothesize that mated female immigrated from nearby fields with a high population density [73].

Lastly, while MD by Shin-Etsu ropes have been widely and effectively used to control *Pectinophora gossypiella* (Saunders) (Gelechiidae) and *Helicoverpa armigera* (Hübner) (Noctuidae) [74], our knowledge about the potential of using aerosol devices for managing fiber crop pests is limited. Kehat and Dunkelblum [74] conducted field experiments against cotton pests in Israel. They tested a polymeric aerosol formulation (i.e., with the aerosol active component stabilized in polymer particles) containing the major sex pheromone component of *Spodoptera littoralis* (Boisduval) (Noctuidae). Despite the results being limited, they are promising.

## 6. Optimization of Aerosol Point Sources, Emission Rates and Deployment

As discussed in the previous chapters, finding a compromise between the deployment of an adequate number of point sources releasing efficacious amounts of pheromone and the cost of the product and its application is a crucial challenge for aerosol adoption. Unfortunately, very few studies have focused on this issue.

It has been shown that Isomate CM MIST formulated with 50% less codlemone (3.5 mg/emission) led to orientation disruption comparable to the standard commercial formulation (7 mg/emission). Decreased periods of dispenser operation (3 and 6 h) and frequency of pheromone emission (30 and 60 min) provided a level of orientation disruption comparable to the current standard protocol of releasing pheromone over a 12-h period on a 15-min cycle, respectively. Overall, altering the aerosol emission frequency from 15 min to 30 or 60 min saves 50% to 75% pheromone without reducing the control efficacy [53]. A 5-year research study carried out in Washington State aimed at comparing the performances of passive Isomate^®^ CM Flex vs. aerosol treatments, Checkmate^®^ Puffer^®^ CM-O or Isomate^®^ CM MIST at 25% of the full rate, for MD of *C. pomonella* showed that aerosol devices did not perform as well, with respect to male catch disruption, as the Isomate^®^ CM Flex dispenser. When testing Isomate^®^ CM MIST technology, it has been observed that a reduction in the hours of pheromone release from 12 to 7 h did not reduce CM catch suppression. Regarding the levels of fruit damage in the Checkmate^®^ Puffer^®^ CM-O and Isomate^®^ CM MIST fields, no significant differences were observed, and a decline in fruit injury was noted in the presence of aerosol devices releasing lower levels of CM pheromone [9]. The manipulation of deployment strategies of aerosol dispensers with respect to wind direction may augment efficacy [45]. Remote-controlled pheromone dispensers are now available (www.semios.com). They are equipped with an array of sensors collecting weather data that can help to take decisions about the best placement for maximum performance and reliability. Combining these data with those from other sources, for instance, camera-equipped traps monitoring pest activity [69], it is possible to generate real-time data streaming that, if needed, activate remotely controlled aerosol dispensers to spray pheromones [75].

## 7. Conclusions and Challenges for Future Research

Overall, our analysis of the literature currently available on the effectiveness of aerosol devices for MD programs highlights key facts and trends to be considered for further research as well as for real-world uses. Strengths and weaknesses of aerosols devices are summarized below:

Strengths:

1. When the pheromone polymerization is slow, as in the case of codlemone, there is strong evidence that the amount of pheromone released per unit could be reduced without compromising the overall efficacy [9,53].
2. Compared to the deployment of passive dispensers, aerosol delivery systems have the advantage of being faster and cheaper to apply.
3. Aerosol devices offer a better protection of the pheromone active ingredient from environmental degradation.
4. Targeting multiple pest species is easier with aerosol devices compared to passive dispensers, pending comparable daily activity of the targeted pests.
5. MD aerosol devices can be programmed to release pheromone for short durations when the target pest is active (e.g., *L. botrana*) [69].
6. Aerosol delivery systems can help to finely tune pheromone release rates over time; this could be important for pests characterized by a low population density during the early season, then growing over time (e.g., the honeydew moth, *Cryptoblabes gnidiella* (Millière) (Pyralidae) [76].
7. Modern digital electronic and information technologies will support the improvement of efficacy by helping the deployment, failure control, and optimization of pheromone release.

Challenges:

1. Borders of aerosol-treated blocks sometimes need to be reinforced either by applying passive pheromone products or additional insecticide treatments.
2. Aerosol pheromone delivery systems are most efficacious when large areas are treated [9].
3. Aerosol field deployment requires a considerable preparatory work to define the installation points, especially in case MD is applied to a set of small properties of irregular geometrical shape; a high degree of coordination between users, technicians, and companies using information technology and modern georeferencing tools is needed to select the best installation site according to the topography and wind direction.
4. Efficacy of aerosol devices can be reduced due to the lack of foliage in the early season [42].
5. MD aerosols are a system with mechanical–electronic technologies so device failure remains a point of weakness.
6. Wind can strongly affect the efficacy of aerosols: sites characterized by strong prevailing winds that vary in intensity will require site-specific deployment strategies to account for this variability.
7. Some MD aerosol devices are still using commercial formulations containing diluents and propellants that are not organic certified.
8. Finally, in some cases, the aerosol devices are also more susceptible to vandalism compared to the passive dispensers.

## Figures and Tables

**Table 1:** Commercial and experimental aerosol devices available or tested so far for field mating disruption of agricultural insect pests.

Aerosol Device	University or Company	Development Level (Experimental/Commercial)	Target Pest	References
Isomate^®^ Mist	Pacific Biocontrol Corporation-USA	commercial	*C. pomonella, G. molesta, A. transitella*	[37]
CheckMate Puffer^®^	Suterra LLC USA	commercial	*C. pomonella, G. molesta, L. botrana, Anarsia lineatella, A. transitella*	[38]
Semios	Semios Technologies Inc. CA	commercial	*Choristoneura rosaceana, Pandemis pyrusana, C. pomonella, G. molesta; A. transitella, A. aurantii*	[39]
NoMate^®^ CM Smart Release	SCENTRY BIOLOGICALS, INC., USA	commercial	*C. pomonella*	[40]
Isomate^®^ CM Mister 1.0	CBC Europe Srl - Italy	commercial	*C. pomonella, Adoxophyes orana;* leafroller species	[41]
MISTER PRO	CBC Europe Srl - Italy	experimental	*L. botrana*	[42]
MSTRS™	Penn State University, USA	experimental	*C. cautella, R. naevana, A. transitella, O. nubilalis*	[30,31,43,44,45]
Neburel^®^	Ecología y Protección Agrícola SL (Valencia, Spain)	experimental	*C. suppressalis*	[20]
Michigan State Microsprayer	Michigan State University, USA	experimental	*P. interpunctella*	[32,46]
